# Nivolumab vs Pembrolizumab for Treatment of US Patients With Platinum-Refractory Recurrent or Metastatic Head and Neck Squamous Cell Carcinoma: A Network Meta-analysis and Cost-effectiveness Analysis

**DOI:** 10.1001/jamanetworkopen.2021.8065

**Published:** 2021-05-06

**Authors:** Rui Pei, Yin Shi, Shuhe Lv, Tingting Dai, Fengyu Zhang, Shao Liu, Bin Wu

**Affiliations:** 1 Department of Pharmacy, Xiangya Hospital, Central South University, Changsha, Hunan, China; 2 The Hunan Institute of Pharmacy Practice and Clinical Research, Changsha, Hunan, China; 3 Institute for Rational and Safe Medication Practices, National Clinical Research Center for Geriatric Disorders, Xiangya Hospital, Central South University, Changsha, Hunan, China; 4 Medical Decision and Economic Group, Department of Pharmacy, South Campus, Ren Ji Hospital, School of Medicine, Shanghai Jiaotong University, Shanghai, China

## Abstract

**Importance:**

Nivolumab and pembrolizumab are approved for treating platinum-refractory recurrent or metastatic head and neck squamous cell carcinoma (R/M HNSCC). Physicians and patients are uncertain which drug is preferable, rendering a cost-effectiveness comparison between them necessary.

**Objective:**

To evaluate the cost-effectiveness of nivolumab vs pembrolizumab in treating platinum-refractory R/M HNSCC.

**Design, Setting, and Participants:**

Both the network meta-analysis and cost-effectiveness analysis included patients from the CheckMate 141 and the KEYNOTE 040 phase 3 randomized clinical trials. The Checkmate 141 trial started on May 1, 2014, with the present analysis based on a September 2017 data cutoff. The KEYNOTE 040 trial started on November 17, 2014, with the present analysis based on a May 15, 2017, data cutoff. A bayesian network meta-analysis that included 856 patients was carried out, and a cost-effectiveness analysis that included 487 patients was conducted by developing a partitioned survival model, both between February and November 2020. The robustness of the model was assessed via 1-way, 2-way, and probabilistic sensitivity analyses; subgroup analyses were included; and scenario analyses were conducted to investigate the associations of dosage adjustment of nivolumab with cost-effectiveness.

**Main Outcomes and Measures:**

Life-years, quality-adjusted life-years (QALYs), overall costs, and incremental cost-effectiveness ratios (ICERs) were measured.

**Results:**

In the cost-effectiveness analysis that included 487 patients, for US health care payers, when nivolumab was administered based on patient weight (3 mg/kg biweekly), at a willingness-to-pay (WTP) threshold of $100 000 per QALY, the probability of nivolumab being cost-effective compared with pembrolizumab was 56%; at a WTP threshold of $150 000 per QALY, the probability was 62%. When nivolumab was administered at a fixed dose of 240 mg biweekly or 480 mg monthly, at a WTP threshold of $100 000 per QALY, the probability of nivolumab being cost-effective was 42% to 45%; at a WTP threshold of $150 000 per QALY, the probability was 52% to 55%.

**Conclusions and Relevance:**

Findings from this network meta-analysis and cost-effectiveness analysis suggest considering both WTP threshold and patient body weight when choosing between nivolumab and pembrolizumab for the treatment of patients with platinum-refractory R/M HNSCC. When the WTP threshold was $100 000 per QALY, for patients weighing less than 72 kg, nivolumab (3 mg/kg, biweekly) was considered cost-effective; otherwise, pembrolizumab was preferable. When the WTP threshold was $150 000 per QALY, nivolumab (3 mg/kg biweekly) was considered cost-effective for patients weighing less than 75 kg; otherwise, fixed-dose nivolumab (240 mg biweekly or 480 mg monthly) provided more cost savings.

## Key Points

**Question:**

Is nivolumab or pembrolizumab more cost-effective for treatment of US patients with platinum-refractory recurrent or metastatic head and neck squamous cell carcinoma?

**Findings:**

In this cost-effectiveness analysis that included 487 patients, when the willingness-to-pay threshold was $100 000 per quality-adjusted life-year, for patients weighing less than 72 kg, nivolumab (3 mg/kg administered biweekly) was considered cost-effective; otherwise, pembrolizumab was preferable. When the willingness-to-pay threshold was $150 000 per quality-adjusted life-year, nivolumab (3 mg/kg biweekly) was considered cost-effective for patients weighing less than 75 kg; otherwise, fixed-dose nivolumab administration (240 mg biweekly, or 480 mg monthly) provided more cost savings.

**Meaning:**

Findings suggest considering both the willingness-to-pay threshold and patient body weight when choosing between nivolumab and pembrolizumab for treating platinum-refractory recurrent or metastatic head and neck squamous cell carcinoma.

## Teaser

This network meta-analysis and cost-effectiveness analysis assesses whether nivolumab or pembrolizumab is more cost-effective for treatment of US patients with platinum-refractory recurrent or metastatic head and neck squamous cell carcinoma (HNSCC).

## Introduction

As the seventh most common cancer, head and neck cancers resulted in 890 000 cases diagnosed and 450 000 deaths worldwide,^1^ including 51 540 new cases and 10 030 deaths in the US in 2018.^2^ Squamous cell carcinoma accounts for more than 90% of head and neck cancers.^3^ Most patients with head and neck squamous cell carcinoma (HNSCC) are diagnosed at the locally advanced stage, and more than half of the cancers will relapse or metastasize.^4^ For patients with recurrent or metastatic HNSCC (R/M HNSCC) that progressed during or after platinum-based chemotherapy, multiple conventional treatment regimens have failed to improve overall survival (OS). This lack of increased OS and the high incidence of adverse events make seeking new treatment options urgent.

Nivolumab and pembrolizumab are humanized immunoglobulin G4 monoclonal antibodies that inhibit programmed cell death 1.^5^ Two phase 3 clinical trials (CheckMate 141 and KEYNOTE 040) have shown a survival advantage of immune checkpoint inhibitors compared with standard-of-care treatment (methotrexate, docetaxel, or cetuximab) for platinum-refractory R/M HNSCC. In the CheckMate 141 trial, the nivolumab group showed improved OS, with a hazard ratio (HR) of 0.68 (95% CI, 0.54-0.86),^6^ and in the KEYNOTE 040 trial, receiving pembrolizumab led to an HR for OS of 0.80 (95%, CI 0.65-0.98).^7^ The improved OS enabled these 2 drugs to stand out and obtain the approval of the US Food and Drug Administration for the treatment of platinum-refractory R/M HNSCC.^8,9^

Along with this compelling clinical performance, the attendant high price has been in the spotlight. Previous studies have shown that, for US health care payers, both nivolumab and pembrolizumab are cost-effective compared with standard-of-care treatment (methotrexate, docetaxel, or cetuximab),^10,11^ whereas few reports have compared the cost-effectiveness of nivolumab vs pembrolizumab.^12^ Thus, this analysis evaluated the cost-effectiveness of nivolumab vs pembrolizumab for the treatment of patients with platinum-refractory R/M HNSCC from the perspective of US health care payers.

## Methods

### Network Meta-analysis

#### Study Selection and Assessment of Risk of Bias

We searched the Cochrane Central Register of Controlled Trials, PubMed, and Embase for eligible publications until September 28, 2020. Meeting abstracts in the American Society of Clinical Oncology and the European Society of Medical Oncology were also reviewed. Details of the study selection are given in the eMethods and eFigure 1 in the Supplement. The risk of bias for clinical trials was assessed in RevMan, version 5.4, according to the guidance provided in the Cochrane handbook.^13^

#### Statistical Analysis

We performed the bayesian network meta-analysis in R, version 4.0.2 (R Project for Statistical Computing), with the gemtc package to obtain the HRs for OS and progression-free survival (PFS) between nivolumab and pembrolizumab. Owing to the dearth of data to evaluate the heterogeneity between trials, a fixed-effects model was chosen for the analysis.^14^

### Cost-effectiveness Analysis

In the cost-effectiveness analysis, nivolumab was compared with pembrolizumab because they are approved immunotherapies in the US for treatment of patients with platinum-refractory R/M HNSCC. A 3% discount rate per year was used for both cost and effectiveness.^15^ We measured life-years, quality-adjusted life-years (QALYs), overall costs, and incremental cost-effectiveness ratios (ICERs) between the treatments. The willingness-to-pay (WTP) threshold was $100 000 to $150 000 per QALY.^16^ This study was conducted and reported following the Consolidated Health Economic Evaluation Reporting Standards (CHEERS) reporting guideline.^17^ This evaluation used no individual patient-level data to inform the analysis and thus does not constitute human subjects research and does not require institutional review board review or exemption or approval by an ethics committee, according to the US Department of Health and Human Services (45 CFR §46).

#### Population and Interventions

The cohort of patients was obtained from CheckMate 141 and KEYNOTE 040 randomized clinical trials, and patient baseline characteristics are given in eTable 1 in the Supplement. The Checkmate 141 trial started on May 1, 2014, and the present analysis was based on a September 2017 data cutoff. The KEYNOTE 040 trial started on November 17, 2014, the present analysis was based on a May 15, 2017 data cutoff. Pembrolizumab was administered at a dosage of 200 mg triweekly; nivolumab was administered at dosage of 3 mg/kg biweekly. The maximum treatment duration for both pembrolizumab and nivolumab was 24 months. When disease progressed or intolerable adverse events occurred, patients received subsequent treatment (eTable 2 in the Supplement) and then received best supportive care until death. Severe adverse events (SAEs; grade ≥3) were based on Common Terminology Criteria for Adverse Events, version 5.0,^18^ and included diarrhea, anemia, stomatitis, mucosal inflammation, neutropenia, hyponatremia, fatigue, and lymphopenia. The treatment discontinuation rate triggered by SAEs in the nivolumab group was 4.2%,^6^ and it was 6% in the pembrolizumab group.^7^ A mean weight of 70 kg and a mean body surface area of 1.86 m^2^ were used to calculate drug dosages.^19^

#### Model Structure

Given the merits of incorporating therapeutic effects over time without calculating transition probabilities,^20^ a 1-month cycle of a partitioned survival model was established using TreeAge Pro 2020 (TreeAge Software) with 3 mutually independent health states: PFS, progressive disease (PD), and death (eFigure 2 in the Supplement). In the model, the proportion of patients in each health state at each time point was determined from OS and PFS curves.^21^ The time horizon was 15 years given that more than 99% of the cohort died, and 5 to 30 years were included in the sensitivity analyses.

#### Effectiveness

To construct the survival model, graphic data from the CheckMate 141 and the KEYNOTE 040 trials were extracted by GetData Graph Digitizer, version 2.26, and time-to-event data were obtained as described in the study by Guyot et al.^22^ Subsequently, with the use of the Akaike Information Criterion and Bayesian Information Criterion, the best-fit parametric models for reconstructed data were chosen among the exponential, Weibull, log-logistic, lognormal, Gompertz, and generalized gamma distributions.^23^ Additional details concerning model fitting are given in eTable 3 and eFigure 3 in the Supplement.

The health utilities were sourced from an updated cost-effectiveness analysis generated by applying a US population preference-weighting algorithm to the EuroQol-5D 3-level health questionnaire data from the CheckMate 141 trial.^11^ Health utilities for KEYNOTE 040 were unavailable; thus, we used those from CheckMate 141 given their similarities. The adverse events associated with utility decrements were obtained from the literature^11,24,25,26,27,28,29,30^ (eTable 4 in the Supplement).

#### Costs

The direct medical costs were covered, including drug acquisition, therapy administration, immunohistochemical test, follow-up, subsequent treatment, best supportive care, terminal care, and SAE management costs. Drug costs were derived from the 2020 mean sale price of the Centers for Medicare & Medicaid Services (Table 1).^6,7,11,19,24,25,26,27,28,29,30,31,32,33,34,35,36,37,38,39,40,41,42,43^ Given the amount of leftover nivolumab resulting from the weight-based administration, a rate of 6.1% for the real-world wastage of nivolumab was considered.^42^ We acquired the costs of administration, immunohistochemical tests, and follow-up from the 2020 Medicare physician fee schedule and Medicare fee-for-service payment.^33,34^ Best supportive care and terminal care costs were estimated based on the Surveillance, Epidemiology, and End Results–Medicare data.^35,36^ The management costs associated with adverse events were sourced from the literature.^11,19,37,38,39,40,41^ All costs were inflated to 2020 US dollars using the Consumer Price Index.^44^

**Table 1.** Basic Parameters Input to the Model and the Ranges of the Sensitivity Analyses^a^

Parameter	Baseline value	Lower limit	Upper limit	Distribution	Source
Lognormal OS survival model of nivolumab	μ = 1.9092; σ = 1.3333	ND	ND	ND	Model fitting
Log-logistic PFS survival model of pembrolizumab	γ = 1.6809; λ = 0.3649	ND	ND	ND	Model fitting
HR for OS (nivolumab vs pembrolizumab)	0.86	0.63	1.17	Lognormal	Network meta-analysis
HR for PFS (nivolumab vs pembrolizumab)	0.91	0.66	1.25	Lognormal	Network meta-analysis
Rate of treatment discontinuation					
Pembrolizumab group	0.06	0.045^b^	0.075^b^	Beta	Cohen et al,^7^ 2019
Nivolumab group	0.042	0.032^b^	0.053^b^	Beta	Ferris et al,^6^ 2019
Drug cost (per month), $					
Pembrolizumab	13 403.73	10 052.80^b^	16 754.67^b^	Gamma	CMS^31^
Nivolumab	11 828.04	8871.03^b^	14 785.05^b^	Gamma	CMS^31^
Cetuximab					
The first cycle	13 519.76	10 139.82^b^	16 899.70^b^	Gamma	CMS^31^
The ensuing cycle	11 756.32	8817.24^b^	14 695.40^b^	Gamma	CMS^31^
Methotrexate	14.88	11.16^b^	18.60^b^	Gamma	CMS^31^
Docetaxel	251.29	188.47^b^	314.11^b^	Gamma	CMS^31^
Paclitaxel	82.14	61.61^b^	102.68^b^	Gamma	CMS^31^
Fluorouracil	30.00	22.50^b^	37.50^b^	Gamma	CMS^31^
Carboplatin	39.45	29.59^b^	49.31^b^	Gamma	CMS^31^
Cisplatin	47.84	35.88^b^	59.80^b^	Gamma	CMS^31^
Afatinib	13 104.00	9828.00^b^	16 380.00^b^	Gamma	UpToDate^32^^c^
Drug administration costs, $					
Chemotherapy infusion					
First hour	142.55	122.39	206.68	Gamma	*CPT* code 96413^33^
Additional hour	30.68	27.00	43.02	Gamma	*CPT* code 96415^33^
Immunohistochemical test	107.19	95.15	151.82	Gamma	*CPT* code 88342^33^
Follow-up cost per month	1443.16	1082.37^b^	1803.95^b^	Gamma	*CPT* code 78816^34^
Best supportive care cost per month	4409.00	2050.00	6861.00	Gamma	Ward et al,^19^ 2017; Enomoto et al,^35^ 2015; Gourin et al,^36^ 2014
Terminal care cost	10 561.00	7920.75^b^	13 201.25^b^	Gamma	Enomoto et al,^35^ 2015
SAE management cost, $^d^					
Pembrolizumab group	611.80	289.66	700.52	Gamma	Haddad et al,^11^ 2020; Ward et al,^19^ 2017; Wong et al,^37^ 2018; Burudpakdee et al,^38^ 2012; Hagiwara et al,^39^ 2013; Swallow et al,^40^ 2018; Wan et al,^41^ 2019
Nivolumab group	902.60	417.87	1206.83	Gamma	Haddad et al,^11^ 2020; Ward et al,^19^ 2017; Wong et al,^37^ 2018; Burudpakdee et al,^38^ 2012; Hagiwara et al,^39^ 2013; Swallow et al,^40^ 2018; Wan et al,^41^ 2019
Health utilities^e^					
Progression-free survival	0.805	0.786	0.824	Beta	Haddad et al,^11^ 2020
Progressed disease	0.746	0.716	0.775	Beta	Haddad et al,^11^ 2020
SAE utility toll^f^					
Pembrolizumab group	0.007	0.004	0.010	Beta	Haddad et al,^11^ 2020; Kohn et al,^24^ 2017; Nafees et al,^25^ 2017; Lloyd et al,^26^ 2008; Zargar et al,^27^ 2018; Tam et al,^28^ 2013; Lee et al,^29^ 2013; Beusterien et al,^30^ 2010
Nivolumab group	0.013	0.007	0.018	Beta	Haddad et al,^11^ 2020; Kohn et al,^24^ 2017; Nafees et al,^25^ 2017; Lloyd et al,^26^ 2008; Zargar et al,^27^ 2018; Tam et al,^28^ 2013; Lee et al,^29^ 2013; Beusterien et al,^30^ 2010
Rate of wastage of nivolumab	0.061	0.046^b^	0.076^b^	Beta	Fukudo et al,^42^ 2020
Body surface area, m^2^	1.86	1.40^b^	2.33^b^	Normal	Ward et al,^19^ 2017
Body weight, kg	70	50	90	Normal	Ward et al,^19^ 2017
Discount rate	0.03	0	0.08	Uniform	Sanders et al,^15^ 2016
Time horizon, mo	180	60	360	Uniform	Tringale et al,^43^ 2018

Abbreviations: CMS, Centers for Medicare & Medicaid Services; *CPT*, *Current Procedural Terminology*; HR, hazard ratio; ND, not determined; OS, overall survival; PFS, progression-free survival; SAE, severe adverse event.

^a^ For additional details, see eTable 4 in the Supplement.

^b^ Variance of plus or minus 25% from baseline values.

^c^ Adjusted with the same discount of the price for pembrolizumab between UpToDate and CMS.

^d^ The mean cost of toxicity weighted by the frequency of occurrence.

^e^ The health utilities are values that vary between 0 and 1 and have no units.

^f^ The mean utility toll of toxicity weighted by the frequency of occurrence.

#### Sensitivity Analyses

To assess the robustness of the model, 1-way deterministic sensitivity analyses using 75 parameters were performed to identify the sensitive factors. Given that the price of nivolumab and pembrolizumab could vary simultaneously, a 2-way sensitivity analysis was also conducted to examine the association of simultaneous changes in these 2 variables with ICERs. Probabilistic sensitivity analyses were performed with 10 000 Monte Carlo simulations, and parameter distributions were determined as proposed by the International Society for Pharmacoeconomics and Outcomes Research and the Society for Medical Decision Making Modeling Good Research Practices Task Force.^45^ Additional details are provided in eTable 4 in the Supplement.

We also conducted subgroup analyses, which are described in the eMethods in the Supplement. Owing to comparable population pharmacokinetics and dose exposure–dose response relationships, the recommended dosage of nivolumab has been adjusted from 3 mg/kg biweekly to 240 mg biweekly or to 480 mg monthly.^46^ Therefore, additional scenario analyses were conducted to investigate any associations with the dosage adjustment of nivolumab.

## Results

### Network Meta-analysis

Through database searching, 446 records were identified, and 2 phase 3 randomized clinical trials (CheckMate 141 and KEYNOTE 040) involving 856 patients were included in the meta-analysis (eFigure 4 in the Supplement). In the CheckMate 141 trial, 361 patients were given nivolumab or standard-of-care treatment; in the KEYNOTE 040, 495 patients were given pembrolizumab or standard-of-care treatment. The risk of bias is presented in eFigure 5 in the Supplement. The network meta-analysis showed that, for the total population, the HR for OS of nivolumab vs pembrolizumab was 0.86 (95% CI, 0.63-1.17) and the HR for PFS was 0.91 (95% CI, 0.66-1.25).

### Cost-effectiveness Analysis

#### Base-Case Analyses

For the total population of 487 patients, pembrolizumab yielded 0.75 QALYs with an overall cost of $121 257. Compared with pembrolizumab, nivolumab improved effectiveness by 0.14 QALYs and increased the overall cost by $11 816, resulting in an ICER of $86 983 per QALY (Table 2).

**Table 2.** Summary of Base-Case Analyses

Factor	Pembrolizumab	Nivolumab	Incremental nivolumab vs pembrolizumab
LYs	1.02	1.21	0.19
QALYs	0.75	0.89	0.14
Drug acquisition cost, $	59 586	60 496	910
Drug administration cost, $	845	1374	529
Subsequent treatment cost, $	11 206	10 134	−1071
Follow-up cost, $	18 528	22 413	3885
Best supportive care cost, $	23 732	33 440	9708
Terminal care cost, $	10 539	10 488	−51
Adverse event cost, $	612	903	291
Immunohistochemical test cost, $	107	107	0
Overall cost, $	125 154	139 356	14 201
Overall cost (discounted), $	121 257	133 073	11 816
ICER, $/LY	NA	NA	62 114
ICER, $/QALY	NA	NA	86 983

Abbreviations: ICER, incremental cost-effectiveness ratio; LYs, life-years; NA, not applicable; QALYs, quality-adjusted life-years.

#### Univariate and Probabilistic Sensitivity Analyses

**Figure 1.**
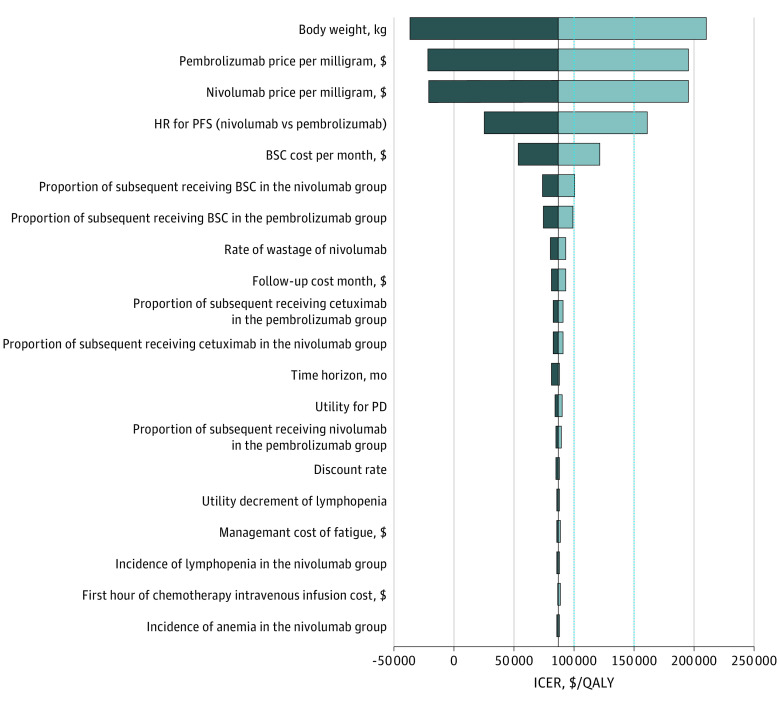
Tornado Diagrams of Univariable Sensitivity Analyses The diagram shows the association of variables with the incremental cost-effectiveness ratio (ICER) of nivolumab vs pembrolizumab in the treatment of platinum-refractory recurrent or metastatic head and neck squamous cell carcinoma. The vertical black line represents the primary result of $86 983 per quality-adjusted life-year (QALY) as the ICER in the base-case analysis. The vertical dotted lines represent the $100 000 and $150 000 per QALY willingness-to-pay thresholds used in the analysis. At the lower limit of the body weight (50 kg), the ICER was −$36 606 per QALY; at the upper limit of the body weight (90 kg), the ICER was $210 572 per QALY. BSC indicates best supportive care; HR, hazard ratio; PD, progressive disease; and PFS, progression-free survival.

The model was particularly sensitive to body weight, the cost of pembrolizumab and nivolumab, and the HR for PFS (nivolumab vs pembrolizumab) (Figure 1). We explored the association of these key factors with the ICER between nivolumab and pembrolizumab. For a WTP threshold of $100 000 per QALY, receipt of nivolumab was beyond the means of patients who weighed more than 72 kg; at a WTP threshold of $150 000 per QALY, pembrolizumab was cost-effective for patients who weighed more than 80 kg (eFigure 6 in the Supplement). For a WTP threshold of $100 000 per QALY, when the cost of pembrolizumab exceeded $49 per mg or the cost of nivolumab was less than $29 per mg or the HR for PFS was higher than 0.85, nivolumab was cost-effective; for a WTP threshold of $150 000 per QALY, when the cost of pembrolizumab exceeded $43 per mg or the cost of nivolumab was less than $33 per mg or the HR for PFS was higher than 0.70, nivolumab was cost-effective; otherwise, pembrolizumab was preferable. A 2-way sensitivity analysis revealed that when the cost of nivolumab was less than $22 per mg, the ICER was lower than $100 000 per QALY regardless of the variability in the price of pembrolizumab between $37 per mg and $63 per mg. When the cost of nivolumab was less than $25 per mg, the ICER was always lower than $150 000 per QALY (eFigure 7 in the Supplement).

**Figure 2.**
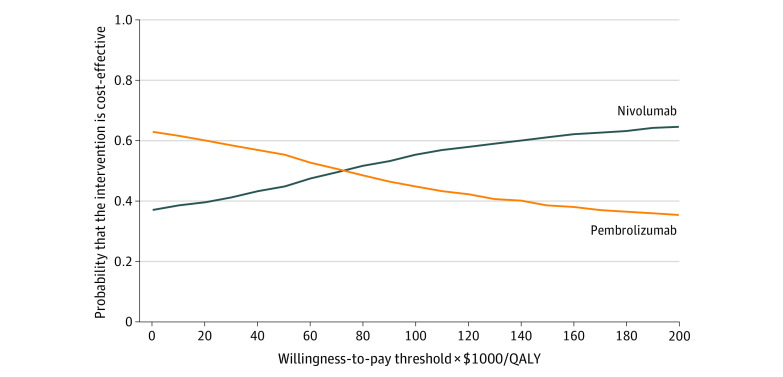
Cost-effectiveness Acceptability Curves Results of probabilistic sensitivity analyses for nivolumab vs pembrolizumab after 10 000 Monte Carlo simulations, which indicates the probability of cost-effectiveness at different willingness-to-pay thresholds based on the uncertainty of the parameters. QALY indicates quality-adjusted life-year.

Probabilistic sensitivity analyses showed that for the total population, compared with pembrolizumab, at the WTP threshold of $100 000 per QALY, the probability of nivolumab being cost-effective was 56%, and for the WTP threshold of $150 000 per QALY, the probability of nivolumab being cost-effective was 62% (Figure 2). The incremental cost-effectiveness scatterplots are shown in eFigure 8 in the Supplement.

#### Subgroup Analyses

For most subgroups, nivolumab performed better in lowering the risk of death, and the ICER of nivolumab vs pembrolizumab ranged from $84 403 per QALY to $89 618 per QALY (Table 3). Solely for the subgroup of patients who were 65 to 75 years of age, pembrolizumab was superior to nivolumab in improving OS, leading to an ICER of $93 725 per QALY for pembrolizumab vs nivolumab. Probabilistic sensitivity analyses indicated an overall trend for subgroups with better survival advantages to have a higher probability of cost-effectiveness.

**Table 3.** Summary of Subgroup Analyses

Subgroup	HR for OS (95% CI)^a^	Change in cost, $^a^	Change in QALYs^a^	ICER, $/QALY	Cost-effectiveness probability of nivolumab, %	
					WTP of $100 000 per QALY	WTP of $150 000 per QALY
Sex						
Male	0.84 (0.58 to 1.22)	13 540	0.15	87 770	56	62
Female	0.99 (0.42 to 2.31)	−444	0.00	NA	51	47
Age, y						
<65	0.68 (0.45 to 1.03)	25 192	0.28	89 454	57	73
≥65 to <75	1.63 (0.84 to 3.17)	−68 555	−0.73	93 725	45	23
≥75^b^	NA	NA	NA	NA	NA	NA
ECOG performance status						
0	0.72 (0.36 to 1.46)	22 697	0.25	89 618	54	62
≥1	0.90 (0.63 to 1.27)	8227	0.10	84 403	54	57
p16 Status						
Positive	0.62 (0.32 to 1.18)	28 411	0.32	88 853	55	70
Negative	0.77 (0.47 to 1.26)	19 173	0.21	89 373	55	64
Previous cetuximab use						
Yes	0.89 (0.60 to 1.32)	9141	0.11	85 234	54	56
No	0.67 (0.41 to 1.11)	25 771	0.29	89 379	56	70
PD-L1						
Positive	0.75 (0.50 to 1.14)	20 638	0.23	89 548	55	66
Negative	0.57 (0.31 to 1.05)	30 641	0.35	88 135	57	74

Abbreviations: ECOG, Eastern Cooperative Oncology Group; HR, hazard ratio; ICER, incremental cost-effectiveness ratio; NA, not available; OS, overall survival; PD-L1, programmed cell death ligand 1; QALYs, quality-adjusted life-years; WTP, willingness to pay.

^a^ HR for OS represents the HR of nivolumab vs pembrolizumab for OS; change in cost and change in QALYs represent the results of nivolumab minus pembrolizumab.

^b^ HR unavailable due to the small sample size in this subgroup.

#### Scenario Analyses

At a dosage of 240 mg biweekly, the overall cost of nivolumab was $137 696, resulting in an ICER of nivolumab vs pembrolizumab of $120 356 per QALY. At 480 mg monthly, the overall cost of nivolumab was $137 015, leading to an ICER of $115 442 per QALY. Probabilistic sensitivity analyses showed that compared with pembrolizumab, for 240 mg biweekly, the probability of nivolumab being cost-effective at a WTP threshold of $100 000 per QALY was 42%; at a WTP threshold of $150 000 per QALY, it was 52%. At 480 mg monthly, the probability of nivolumab being cost-effective was 45%; at a WTP threshold of $150 000 per QALY, it was 55%.

## Discussion

Health care spending associated with head and neck cancer was 3.64 billion in 2010 and projected to be 4.34 to 5.46 billion in 2020.^47^ Given escalating health care costs, concern regarding value-based oncology is warranted, with nivolumab and pembrolizumab, the leading therapeutics in the immunotherapy pipeline, garnering much attention. Because both have been approved to treat platinum-refractory R/M HNSCC, physicians and patients face a dilemma in determining which is preferable, rendering necessary a cost-effectiveness comparison.

Our analysis found that, for the total population, compared with pembrolizumab, nivolumab improved effectiveness by 0.14 QALYs and increased the overall cost by $11 816, leading to an ICER of $86 983 per QALY. The additional cost associated with nivolumab stemmed primarily from follow-up costs and best supportive care costs, which came with prolonged survival. Sensitivity analyses indicated that body weight was the most sensitive factor, suggesting that the choice between nivolumab and pembrolizumab could be made based on the patient’s weight. At a WTP threshold of $100 000 per QALY, nivolumab was cost-effective for patients weighing less than 72 kg, and at a WTP threshold of $150 000 per QALY, pembrolizumab was preferable for those weighing more than 80 kg. The costs of pembrolizumab and nivolumab were also important factors. If the cost of pembrolizumab decreased to $49 per mg, it was cost-effective at a WTP threshold of $100 000 per QALY. If the cost decreased to $43 per mg, it was more cost-effective than nivolumab at the WTP threshold of $150 000 per QALY. If the cost increased by 10%, the ICER converged to zero, indicating that the overall costs of nivolumab and pembrolizumab were nearly identical. When the cost of nivolumab increased above $29 per mg, for patients with a WTP threshold of $100 000 per QALY, nivolumab was not cost-effective. When the cost rose above $33 per mg, for patients with a WTP threshold of $150 000 per QALY, nivolumab was less appealing than pembrolizumab. When the cost of nivolumab dropped below $23 per mg, the ICER was close to zero. The HR for PFS (nivolumab vs pembrolizumab) was another key factor. When the HR for PFS was 0.85, the ICER of nivolumab vs pembrolizumab was $100 000 per QALY; when the HR was 0.70, the ICER was $150 000 per QALY.

We also investigated the administration of nivolumab at a fixed dose. At 240 mg biweekly or 480 mg monthly, the ICER of nivolumab vs pembrolizumab was $120 356 per QALY and $115 442 per QALY, respectively, both of which were higher than $86 983 per QALY, the ICER of nivolumab vs pembrolizumab at a dosage based on patient weight. This finding suggests that dose adjustments, although helpful for reducing waste and facilitating administration, potentially impose a heavier financial burden on patients with low body weight. At a body weight of 75 kg, the weight-based and fixed-dose administration costs were equivalent. Thus, for patients weighing less than 75 kg, the weight-based dosage was associated with greater cost savings; otherwise, a fixed dosage was preferred. Compared with the weight-based dosage of nivolumab, the probability of a fixed dose of either 240 mg biweekly or 480 mg monthly being cost-effective was 30% and 33%, respectively.

A study by Yeh and Guddati^12^ compared the cost-effectiveness of nivolumab vs pembrolizumab in the treatment of R/M HNSCC, yielding the ICERs of nivolumab vs standard of care and pembrolizumab vs standard of care of $484 184 per QALY and $856 173 per QALY, respectively. The differences between the results of their study and ours may be explained as follows. First, their study was an indirect comparison of 2 immunotherapies with a common combined standard. Second, no model was constructed in their study, and only 24 months of QALYs were considered, which would underestimate the benefits of immunotherapies given their delayed therapeutic effects. Third, the initial health utility was unclear, the disutilities associated with SAEs were not described, and the PD health state was given a utility of zero, which largely contributed to the inconsistent results between our studies. Fourth, no costs for subsequent treatment, follow-up, terminal care, and best supportive care were considered. By contrast, our study conducted a direct comparison of the 2 immunotherapies by using a meta-analysis approach, developing a 15-year partitioned survival model, and including 75 variables to fully reflect the cost and effectiveness of the immunotherapies.

This study is, to our knowledge, the first to investigate the cost-effectiveness of pembrolizumab vs different dosages of nivolumab for treatment of platinum-refractory R/M HNSCC. The clinical implications of this study warrant discussion. For US patients with platinum-refractory R/M HNSCC, it may not be wise to completely abandon weight-based dosing in favor of a fixed dose, and dosing regimens should be individualized. We recommend taking into consideration both the WTP threshold and patient weight to make an optimal clinical decision. Provided that the WTP threshold is $100 000 per QALY, for patients weighing less than 72 kg, nivolumab, 3 mg/kg, administered biweekly is most cost-effective; otherwise, pembrolizumab is preferable. For a WTP threshold of $150 000 per QALY, nivolumab, 3 mg/kg, administered biweekly is cost-effective for patients weighing less than 75 kg; otherwise, a fixed-dose of nivolumab (either 240 mg biweekly or 480 mg monthly) provides the greatest cost savings.

### Limitations

This study had some limitations. First, although the CheckMate 141 and the KEYNOTE 040 trials are similarly focused on platinum-refractory R/M HNSCC, there are some disparities between them.^48^ The potential bias in these 2 trials is difficult to determine in the absence of the original patient data; thus, the results of the present study should be interpreted cautiously. Second, owing to the dearth of data on all-cause SAEs for pembrolizumab, we used the available treatment-related SAEs for nivolumab and pembrolizumab. Third, categorization of the expression of programmed death ligand 1 differed between the 2 trials. In KEYNOTE 040, the positive or negative expression was categorized as a combined positive score, whereas in CheckMate 141, the expression was categorized as a tumor proportion score. Because HRs for PFS in the various subgroups were unavailable, we used PFS data for the total population. In addition, we assumed that the incidence of SAEs and the percentages of subsequent treatment in the subgroups were the same as those in the total population. Moreover, the small sample size in the subgroups decreased the robustness of our results. Fourth, owing to the absence of utility values for pembrolizumab, we used the values of nivolumab for pembrolizumab given their similarities; our sensitivity analysis indicated that health utilities would not greatly impact the results. Fifth, the face validation of the model was judged by experts, including model structure, assumptions, data sources, analyses, and results. All the uncertainties suggested by the experts were included in the sensitivity analyses. Because pembrolizumab and nivolumab are relatively new for the treatment of patients with R/M HNSCC, long-term observational data were unavailable to externally validate the extrapolation of the models; however, our results did not appear to be particularly sensitive to the extrapolated parameter functions. Sixth, in sensitivity analyses, for variables missing the range of variation, a uniform variance of 25% above and below baseline values was assumed. Although this method is commonly used in economic evaluations, this range may have been inaccurate for some variables. Seventh, given disparities in cost inputs and payer perspectives, our results may not be generalizable to other geographic regions.^49^

## Conclusions

This network meta-analysis and cost-effectiveness analysis for US patients with platinum-refractory R/M HNSCC found that the optimal treatment choice between nivolumab and pembrolizumab may be best decided by considering both the WTP threshold and the patient’s weight. Given a WTP threshold of $100 000 per QALY, for patients weighing less than 72 kg, nivolumab, 3 mg/kg, administered biweekly was most cost-effective; otherwise, pembrolizumab was preferable. Given a WTP of $150 000 per QALY, nivolumab, 3 mg/kg, administered biweekly was most cost-effective for patients weighing less than 75 kg; otherwise, fixed-dose nivolumab (either 240 mg biweekly or 480 mg monthly) provided the greater cost savings.

## Notes

Supplement. **eTable 1.** Patient Baseline Characteristics **eTable 2.** Summary of Subsequent Therapy **eTable 3.** AIC Scores and BIC Scores for Parametric Models **eTable 4.** Model Parameters: Baseline Values, Ranges, and Distributions for Sensitivity Analyses **eFigure 1.** Flowchart of Study Selection **eFigure 2.** Partitioned Survival Model **eFigure 3.** Model Fitting Analysis **eFigure 4.** Model Schematic for Network Meta-analysis **eFigure 5.** Risk of Bias Summary **eFigure 6.** Impacts of Key Factors on Incremental Cost-effectiveness Ratio **eFigure 7.** Results of Two-Way Sensitivity Analyses **eFigure 8.** Incremental Cost-effectiveness Scatter Plot (Nivolumab vs Pembrolizumab) eMethods. eReferences.
