# Supplementary material for: Nivolumab vs Pembrolizumab for Treatment of US Patients With Platinum-Refractory Recurrent or Metastatic Head and Neck Squamous Cell Carcinoma: A Network Meta-analysis and Cost-effectiveness Analysis
Source: JAMA Netw Open. 2021 May 6;4(5):e218065. doi: 10.1001/jamanetworkopen.2021.8065 (PMC8103222; doi:10.1001/jamanetworkopen.2021.8065)
Supplement: Supplement. — eTable 1. Patient Baseline Characteristics eTable 2. Summary of Subsequent Therapy eTable 3. AIC Scores and BIC Scores for Parametric Models eTable 4. Model Parameters: Baseline Values, Ranges, and Distributions for Sensitivity Analyses eFigure 1. Flowchart of Study Selection eFigure 2. Partitioned Survival Model eFigure 3. Model Fitting Analysis eFigure 4. Model Schematic for Network Meta-analysis eFigure 5. Risk of Bias Summary eFigure 6. Impacts of Key Factors on Incremental Cost-effectiveness Ratio eFigure 7. Results of Two-Way Sensitivity Analyses eFigure 8. Incremental Cost-effectiveness Scatter Plot (Nivolumab vs Pembrolizumab) eMethods. eReferences. [file jamanetwopen-e218065-s001.pdf]

Supplementary Online Content

Pei R, Shi Y, Lv S, et al. Nivolumab vs pembrolizumab for treatment of US patients with platinum-refractory recurrent or metastatic head and neck squamous cell carcinoma: a network meta-analysis and cost-effectiveness analysis. *JAMA Netw Open*. 2021;4(4):e218065. doi:10.1001/jamanetworkopen.2021.8065

eTable 1. Patient Baseline Characteristics

eTable 2. Summary of Subsequent Therapy

eTable 3. AIC Scores and BIC Scores for Parametric Models

eTable 4. Model Parameters: Baseline Values, Ranges, and Distributions for Sensitivity Analyses

eFigure 1. Flowchart of Study Selection

eFigure 2. Partitioned Survival Model

eFigure 3. Model Fitting Analysis

eFigure 4. Model Schematic for Network Meta-analysis

eFigure 5. Risk of Bias Summary

eFigure 6. Impacts of Key Factors on Incremental Cost-effectiveness Ratio

eFigure 7. Results of Two-Way Sensitivity Analyses

eFigure 8. Incremental Cost-effectiveness Scatter Plot (Nivolumab vs Pembrolizumab)

eMethods.

eReferences.

This supplementary material has been provided by the authors to give readers additional information about their work.

eTable 1. Patient Baseline Characteristics

**Characteristic**	**Pembrolizumab group**	**Nivolumab group**
Median Age (range)—years	60 (55-66)	59 (29–83)
Male sex	84%	82%
ECOG performance status score		
0	29%	20%
1	71%	79%
≥2	0	<1%
p16 status positive	25%	26%
Previous cetuximab use	59%	62.5%
PD-L1*		
Negative	20%	30%
Positive	79%	37%
Missing/ Not quantifiable	<1%	33%

Abbreviation: ECOG=Eastern Cooperative Oncology Group. PD-L1=Programmed cell death-ligand 1.

* PD-L1 expression was categorized based on different indicators. In the KEYNOTE 040 (Pembrolizumab group), PD-L1 positive/negative expression was categorized by the combined positive score, and it was categorized by the tumor proportion score in the CheckMate 141 (Nivolumab group).

© 2021 Pei R et al. *JAMA Network Open*.

eTable 2. Summary of Subsequent Therapy

	**Pembrolizumab group**	**Nivolumab group**
Proportion of patients receiving subsequent treatment	34.0%	29.6%
Duration of treatment (months)	2.33	2.33
Frequency of treatments		
Cetuximab	8.1%	7.7%
Afatinib	1.2%	-
Pembrolizumab	0.5%	0.3%
Nivolumab	4.0%	0.7%
Docetaxel	3.4%	3.4%
Methotrexate	5.1%	5.7%
Paclitaxel	6.4%	6.4%
Platinum (carboplatin / cisplatin)	4.0%	4.0%
5-Fluorouracil	1.3%	1.3%

* The details of subsequent therapy in the nivolumab group were sourced from the supplementary materials of Haddad R, et al., 2020 [1], an updated cost-effectiveness analysis regarding recurrent or metastatic head-and-neck squamous cell carcinoma (R/M HNSCC). The details of subsequent therapy in the pembrolizumab group were assumed based on the information provided in the appendix of the KEYNOTE 040 trial [2], plus the details in the nivolumab group to keep a balance between these two drugs.

© 2021 Pei R et al. *JAMA Network Open*.

eTable 3. AIC Scores and BIC Scores for Parametric Models

**Parametric models**	**OS of nivolumab**		**PFS of nivolumab**		**OS of pembrolizumab**		**PFS of pembrolizumab**	
	**AIC**	**BIC**	**AIC**	**BIC**	**AIC**	**BIC**	**AIC**	**BIC**
Exponential	1440.591	1444.071	911.175	914.655	1256.133	1259.643	1146.021	1149.531
Log-logistic	1424.965	1431.926	**848.375**	**855.336**	1253.418	1260.437	**1083.503**	**1090.522**
Weibull	1438.245	1445.206	905.534	912.496	1258.131	1265.150	1147.227	1154.246
Lognormal	**1422.746**	**1429.707**	855.395	862.356	**1250.566**	**1257.584**	1093.603	1100.622
Gompertz	1428.948	1435.909	911.497	918.459	1256.891	1263.910	1122.629	1129.647
Generalized gamma	1424.982	1435.424	861.873	872.315	1252.508	1263.036	1100.293	1110.821

Abbreviation: AIC= Akaike information criterion; BIC= Bayesian information criterion; OS=Overall survival; PFS= Progression-free survival.

© 2021 Pei R et al. *JAMA Network Open*.

eTable 4. Model Parameters: Baseline Values, Ranges, and Distributions for Sensitivity Analyses

**Variables**	**Baseline value**	**DSA range**		**PSA distribution (parameters)**	**References**
		**Lower limit**	**Upper limit**		
Lognormal OS survival model of nivolumab	μ = 1.9092<br>σ = 1.3333	ND	ND	ND	Model fitting
Log-logistic PFS survival model of pembrolizumab	γ = 1.6809<br>λ = 0.3649	ND	ND	ND	Model fitting
HR for OS (Nivolumab vs. Pembrolizumab)	0.86	0.63	1.17	Lognormal (-0.15, 0.30)	Network meta-analysis
HR for PFS (Nivolumab vs. Pembrolizumab)	0.91	0.66	1.25	Lognormal (-0.09, 0.31)	Network meta-analysis
**Drug cost, $/mg**					
Pembrolizumab	50.2640	37.6980*	62.8300*	Gamma (64, 1.27)	CMS [3]
Nivolumab	28.1620	21.1215*	35.2025*	Gamma (64, 2.27)	CMS [3]
Cetuximab	6.3206	4.7405*	7.9008*	Gamma (64, 10.13)	CMS [3]
Methotrexate	0.0500	0.0375*	0.0625*	Gamma (64, 1280.00)	CMS [3]
Docetaxel	0.9650	0.7238*	1.2063*	Gamma (64, 66.32)	CMS [3]
Paclitaxel	0.1380	0.1035*	0.1725*	Gamma (64, 463.77)	CMS [3]
5-fluorouracil	0.0030	0.0023*	0.0038*	Gamma (64, 21164.02)	CMS [3]

© 2021 Pei R et al. *JAMA Network Open*.

Carboplatin	0.0538	0.0403*	0.0672*	Gamma (64, 1190.03)	CMS [3]
Cisplatin	0.1929	0.1447*	0.2411*	Gamma (64, 331.78)	CMS [3]
Afatinib	10.9200	8.1900*	13.6500*	Gamma (64, 5.86)	Up to date [4] [a]
**Drug administration costs, $**					
First hour - chemotherapy infusion	142.55	122.39	206.68	Gamma (59.85, 0.37)	CPT 96413 [5]
Additional hour - chemotherapy infusion	30.68	27.00	43.02	Gamma (80.58, 2.30)	CPT 96415 [5]
Immunohistochemical test	107.19	95.15	151.82	Gamma (111.21, 0.96)	CPT 88342 [5]
Follow up cost per month	1443.16	1082.37*	1803.95*	Gamma (64, 0.0443)	CPT 78816 [6,7]
Best supportive care cost per month	4409	2050	6861	Gamma (13.44, 0.0030)	[8–10]
Terminal care cost	10561	7920*	13201*	Gamma (64, 0.0061)	[8]
**SAEs cost, $**					
Diarrhea	18065	7755	20561	Gamma (19.31, 0.0014)	[11,1]
Anemia	22168	7788	23392	Gamma (34.56, 0.0019)	[11,1]
Fatigue	9316	3821	18793	Gamma (7.26, 0.0008)	[1,12]
Hyponatremia	7210	5407*	9013*	Gamma (53.47, 0.0074)	[1]
Neutropenia/ Neutrophil count decreased	18799	12654	20165	Gamma (40.68, 0.0023)	[11,1]

© 2021 Pei R et al. *JAMA Network Open*.

Stomatitis	19861	10476	24665	Gamma (26.74, 0.0014)	[11,1]
Mucosal inflammation	19861	10476	24665	Gamma (26.74, 0.0014)	[11,1]
Lymphopenia/ Lymphocyte count decreased	18799	10457	20165	Gamma (43.65, 0.0025)	Assumed to be the same as leukopenia [11,1]
**Utility**					
Progression-free survival	0.805	0.786	0.824	Beta (1399.36, 338.98)	[1]
Progressed disease	0.746	0.716	0.775	Beta (648.98, 220.96)	[1]
**SAEs utility toll**					
Diarrhea	0.17	0.12	0.22	Beta (38.21, 186.55)	[13,14]
Anemia	0.25	0.12	0.31	Beta (18.49, 60.19)	[15,16]
Fatigue	0.11	0.08	0.14	Beta (47.75, 386.36)	[14]
Neutropenia/ Neutrophil count decreased	0.35	0.15	0.50	Beta (8.99, 18.67)	[13]
Hyponatremia	0.19	0.14*	0.24*	Beta (51.65, 220.19)	[1]
Stomatitis	0.22	0.16	0.44	Beta (17.27, 54.69)	[16,17]
Mucosal inflammation	0.22	0.16	0.44	Beta (17.27, 54.69)	Assumed to be the same as stomatitis
Lymphopenia/ Lymphocyte count decreased	0.35	0.15	0.50	Beta (8.99, 18.67)	Assumed to be the same as neutropenia
**Incidence of SAEs**					

**Pembrolizumab group**					
Diarrhea	0.016	0.012*	0.020*	Beta (62.96, 3872.04)	[2]
Anemia	0.004	0.003*	0.005*	Beta (63.74, 15871.26)	[2]
Stomatitis	0.004	0.003*	0.005*	Beta (63.74, 15871.26)	[2]
Mucosal inflammation	0.004	0.003*	0.005*	Beta (63.74, 15871.26)	[2]
Neutrophil count decreased	0.004	0.003*	0.005*	Beta (63.74, 15871.26)	[2]
**Nivolumab group**					
Anemia	0.013	0.010*	0.016*	Beta (63.16, 4794.92)	[18]
Fatigue	0.025	0.019*	0.031*	Beta (62.38, 2432.62)	[18]
Hyponatremia	0.008	0.006*	0.010*	Beta (63.48, 7871.52)	[18]
Stomatitis	0.004	0.003*	0.005*	Beta (63.74, 15871.26)	[18]
Lymphopenia/ Lymphocyte count decreased	0.013	0.010*	0.016*	Beta (63.16, 4794.92)	[18]
**Rate of treatment discontinuation**					
Pembrolizumab group	0.060	0.045*	0.075*	Beta (60.10, 941.57)	[2]
Nivolumab group	0.042	0.032*	0.053*	Beta (61.27, 1397.54)	[19]
**Proportion of subsequent therapy**					
**Pembrolizumab group**					

© 2021 Pei R et al. *JAMA Network Open*.

Cetuximab	0.081	0.061*	0.101*	Beta (58.74, 666.39)	[2]
Afatinib	0.012	0.009*	0.015*	Beta (63.22, 5205.11)	[2]
Pembrolizumab	0.005	0.003*	0.007*	Beta (63.68, 12671.33)	[2]
Nivolumab	0.040	0.030*	0.050*	Beta (61.4, 1473.60)	[2]
Docetaxel	0.034	0.026*	0.043*	Beta (61.79, 1755.56)	[2]
Methotrexate	0.051	0.038*	0.064*	Beta (60.68, 1129.22)	[2]
Paclitaxel	0.064	0.048*	0.080*	Beta (59.84, 875.16)	[2]
Platinum (Carboplatin/ Cisplatin) [b]	0.040	0.030*	0.050*	Beta (61.40, 1473.60)	[2]
5-Fluorouracil	0.013	0.009*	0.016*	Beta (63.16, 4794.92)	[2]
**Nivolumab group**					
Cetuximab	0.077	0.058*	0.096*	Beta (59.00, 707.17)	[1]
Pembrolizumab	0.003	0.002*	0.004*	Beta (63.81, 21204.53)	[1]
Nivolumab	0.007	0.005*	0.009*	Beta (63.55, 9014.31)	[1]
Docetaxel	0.034	0.025*	0.043*	Beta (61.79, 1755.56)	[1]
Methotrexate	0.057	0.042*	0.072*	Beta (60.29, 997.51)	[1]
Paclitaxel	0.064	0.048*	0.080*	Beta (59.84, 875.16)	[1]
Platinum (Carboplatin/ Cisplatin) [b]	0.040	0.030*	0.050*	Beta (61.40, 1473.60)	[1]

© 2021 Pei R et al. *JAMA Network Open*.

5-Fluorouracil	0.013	0.009*	0.017*	Beta (63.16, 4794.92)	[1]
Rate of wastage of nivolumab	0.061	0.046*	0.076*	Beta (60.04, 924.15)	[20]
Body surface area, m^2	1.86	1.40*	2.33*	Normal (1.86, 0.23)	[10]
Body weight, kg	70	50	90	Normal (70, 10)	[10]
Discount rate	0.03	0	0.08	Uniform	[21]
Time horizon, mo	180	60	360	Uniform	[22]

Abbreviations: CMS= Centers for Medicare & Medicaid services; *CPT= Current procedural terminology*; DSA= Deterministic sensitivity analysis; HR= Hazard ratio; ND= Not determined; OS= Overall survival; PFS= Progression-free survival; PSA= Probabilistic sensitivity analysis; SAE= Severe adverse event.

* Variance of ±25% from baseline values.

[a] Adjusted with the same discount of the price for pembrolizumab between UpToDate and CMS.

[b] Carboplatin and cisplatin were combined because their differences in the drug acquisition costs and administration costs are negligible.

© 2021 Pei R et al. *JAMA Network Open*.

eFigure 1. Flowchart of Study Selection

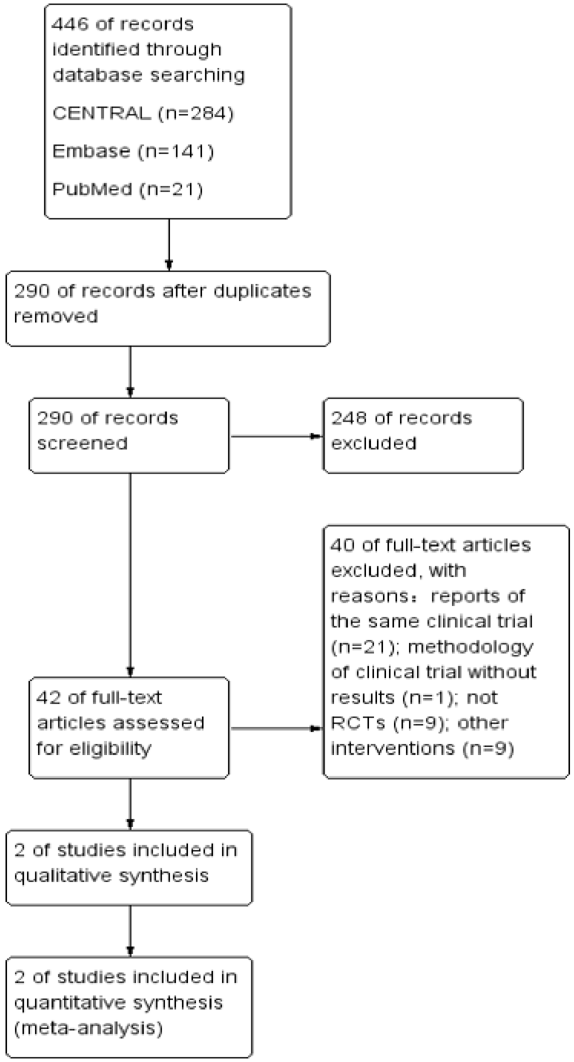

© 2021 Pei R et al. *JAMA Network Open*.

eFigure 2. Partitioned Survival Model.

In the partitioned survival model, PFS and OS curves were utilized to estimate the proportion of patients who were in different health states (PFS, PD or dead) at different time points. OS= Overall survival; PFS= Progression-free survival; PD= Progressive disease.

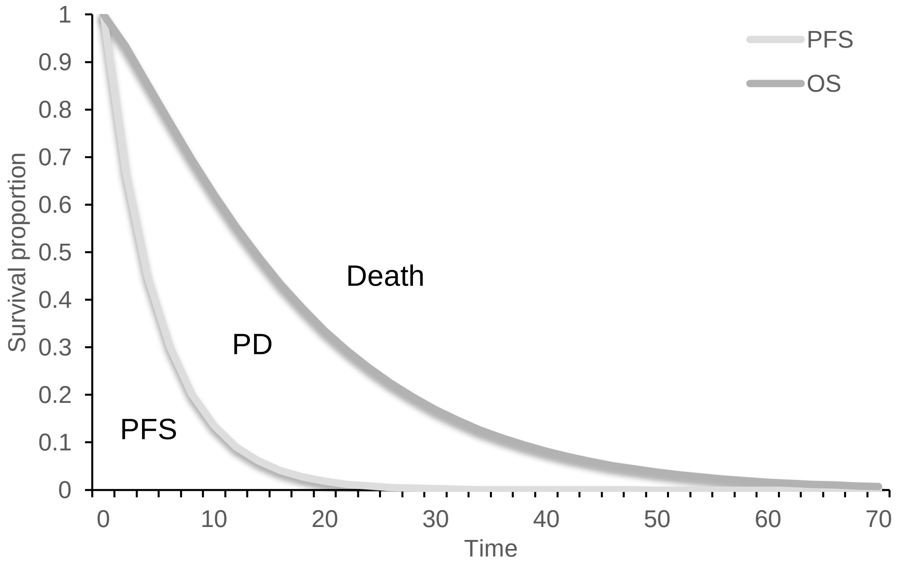

© 2021 Pei R et al. *JAMA Network Open*.

eFigure 3. Model Fitting Analysis

To obtain the best model fit, the following investigations were carried out using pembrolizumab or nivolumab as the model fit baseline, respectively. Abbreviation: AIC= Akaike information criterion; BIC= Bayesian information criterion; HRs=Hazard ratios; OS=Overall survival; PFS= Progression-free survival; KM= Kaplan-Meier.

(1) Pembrolizumab was used as the model fitting baseline. Based on AIC and BIC (eTable 3), lognormal was chosen to fit the OS KM of pembrolizumab, and log-logistic was used to fit the PFS KM of pembrolizumab, and the curves of nivolumab were obtained via the HRs for OS or PFS between nivolumab and pembrolizumab.

A Model-fitted versus original KM curves for pembrolizumab.

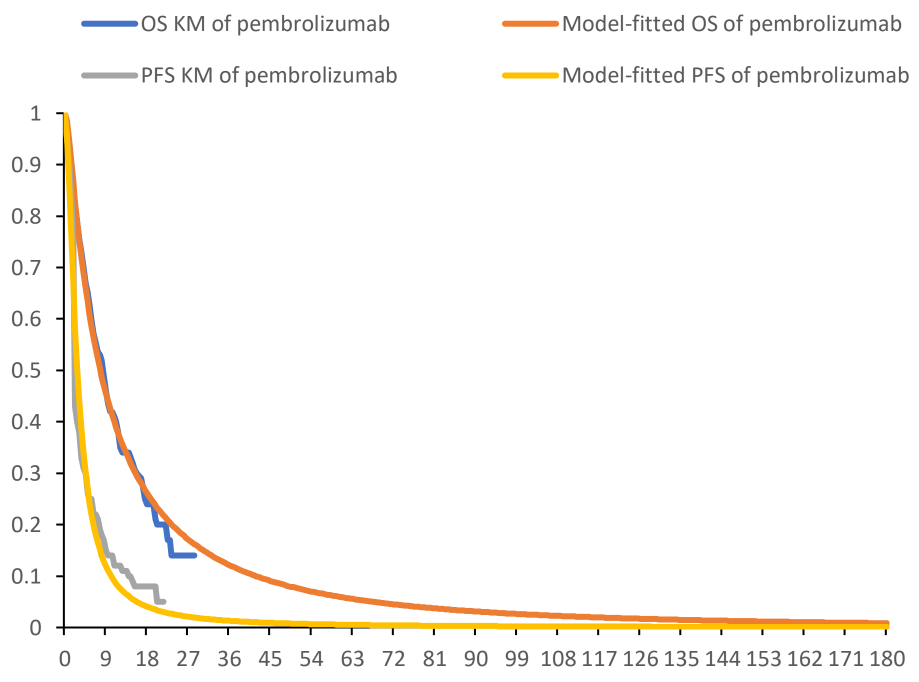

© 2021 Pei R et al. *JAMA Network Open*.

B Model-fitted versus original KM curves for nivolumab.

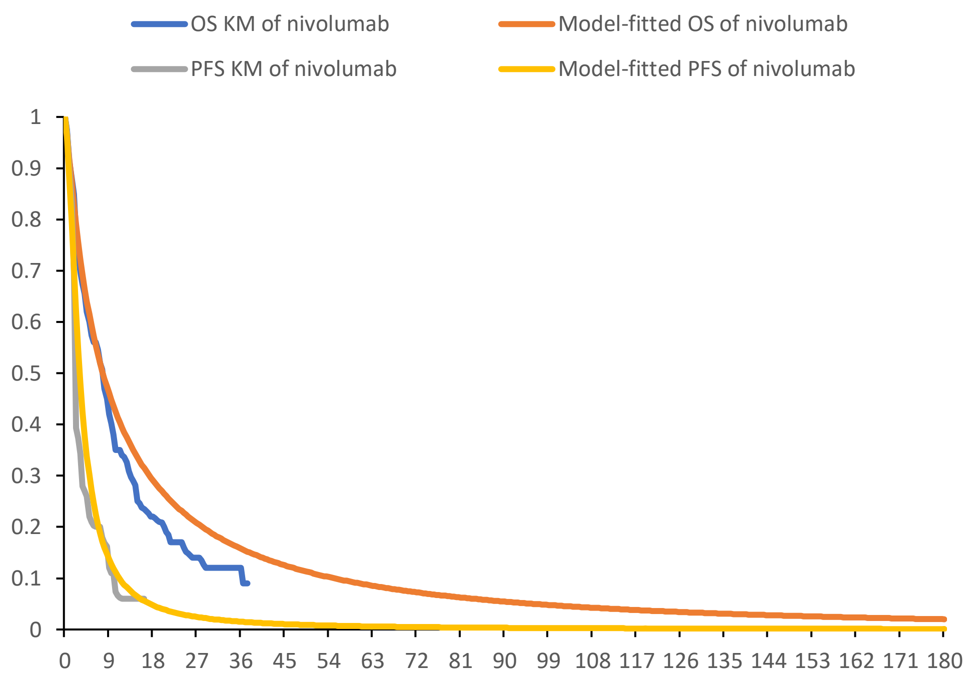

(2) Nivolumab was used as the model fitting baseline. Based on AIC and BIC (eTable 3), lognormal was chosen to fit the OS KM of nivolumab, and log-logistic was used to fit the PFS KM of nivolumab, and the curves of pembrolizumab were obtained via the HRs for OS or PFS between nivolumab and pembrolizumab.

A Model-fitted versus original KM curves for nivolumab.

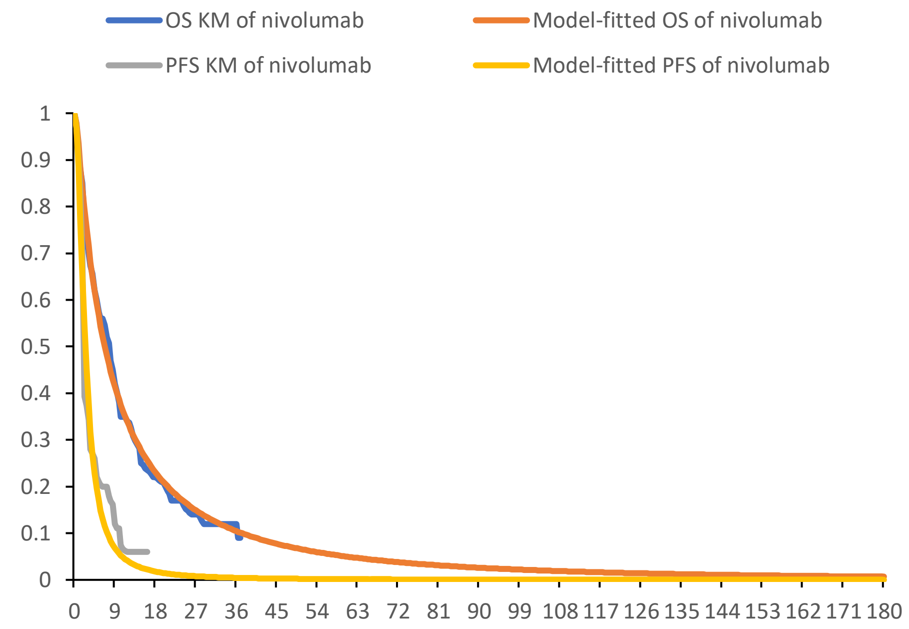

© 2021 Pei R et al. *JAMA Network Open*.

B Model-fitted versus original KM curves for pembrolizumab.

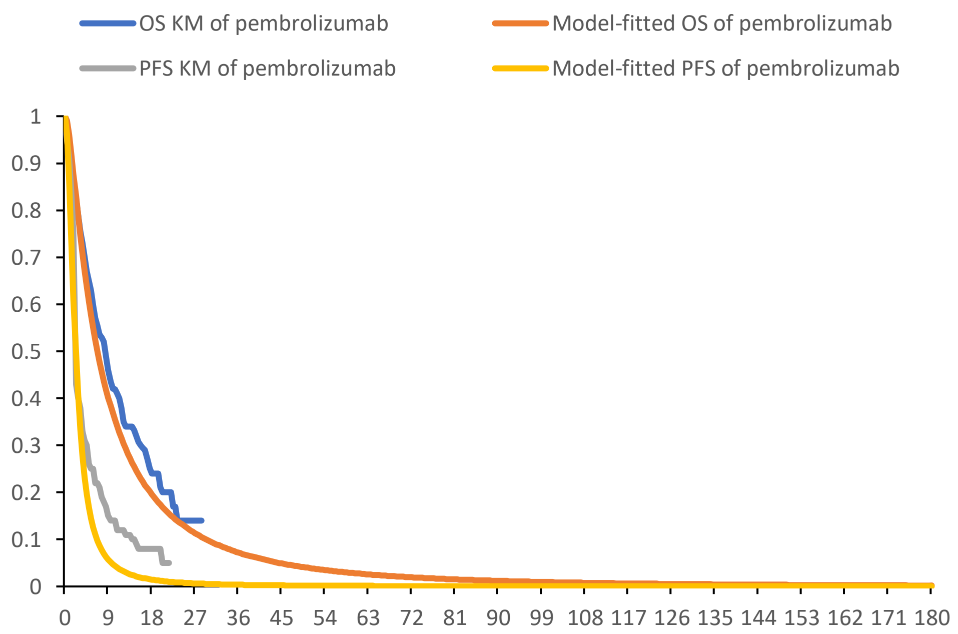

(3) The curves with the more mature follow-up time in both groups were used as the baseline for model fitting. For the OS curves, lognormal was chosen to fit the OS KM of the nivolumab group with longer follow-up, and the OS curve of pembrolizumab was developed by virtue of the HR for OS between nivolumab and pembrolizumab; for the PFS curves, since more mature PFS KM for nivolumab were not available, we fitted the PFS KM for pembrolizumab with the log-logistic model, and then obtained the PFS curve of nivolumab via the HR for PFS.

© 2021 Pei R et al. *JAMA Network Open*.

A Model-fitted versus original KM curves for nivolumab.

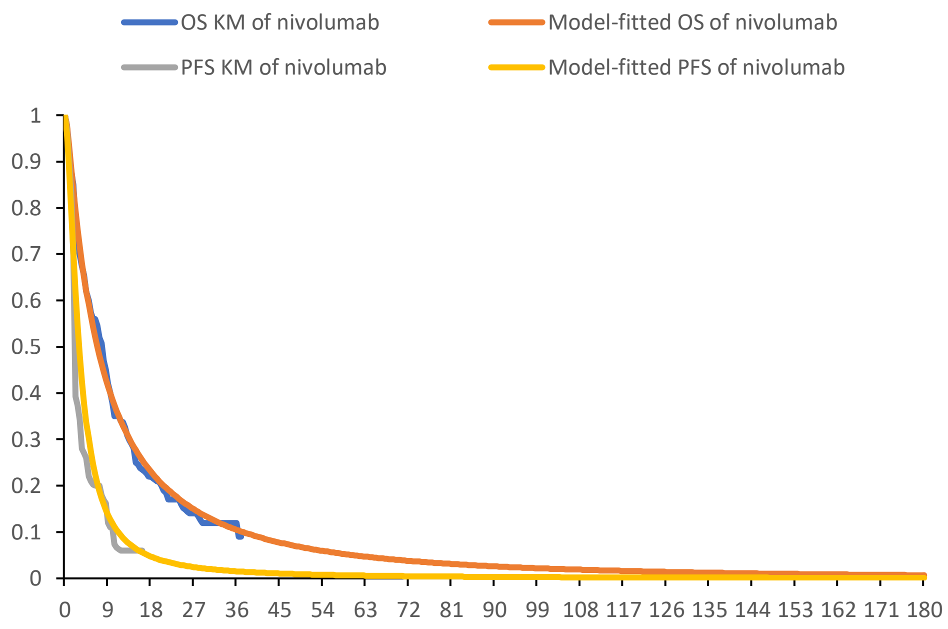

© 2021 Pei R et al. *JAMA Network Open*.

B Model-fitted versus original KM curves for pembrolizumab.

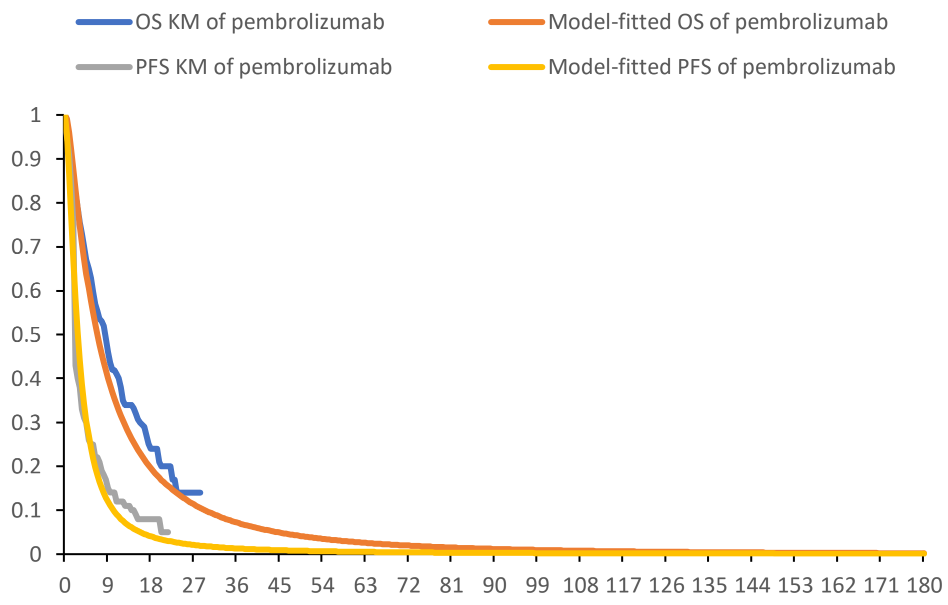

In summary, we found that the model fit based on the curve with more mature follow-up time in each group was relatively better, thus, the third approach was used in our final analysis, namely, for the OS curves, lognormal was chosen to fit the OS KM of the nivolumab group with longer follow-up, and the OS curve of pembrolizumab was developed by virtue of the HR for OS between nivolumab and pembrolizumab; for the PFS curves, since more mature PFS KM for nivolumab were not available, we fitted the PFS KM for pembrolizumab with the log-logistic model, and then obtained the PFS curve of nivolumab via the HR for PFS.

eFigure 4. Model Schematic for Network Meta-analysis

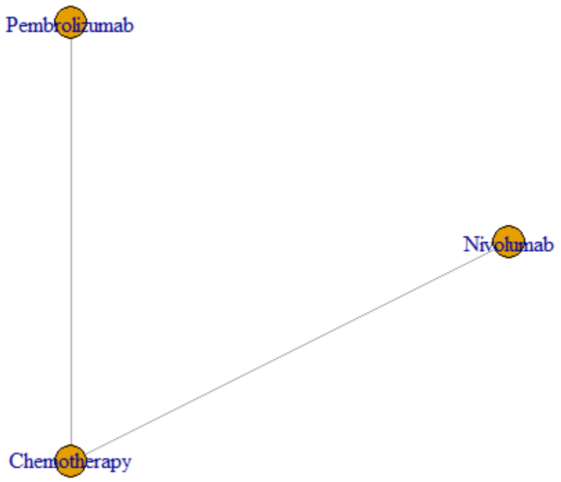

eFigure 5. Risk of Bias Summary

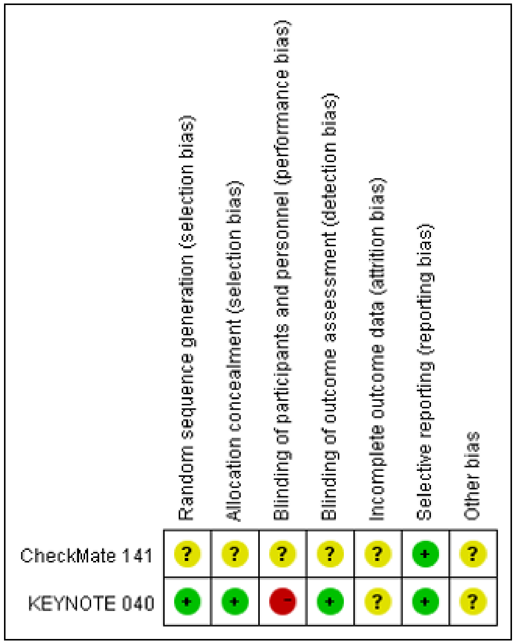

KEYNOTE 040 was judged at low risk in random sequence generation and allocation concealment due to the using of computerized randomization. Checkmate 141 was also a randomized controlled trial, while it did not specify the method of randomization, thus the risk was unclear. In the KEYNOTE 040, given neither patients nor investigators were masked to group assignment, the performance bias was categorized as at high risk, while the outcome was assessed by masked, independent central review, so the detection bias was considered as at low risk. The blinding was not mentioned in the Checkmate 141, so it was classified as at unclear risk for performance bias and detection bias. Two studies were considered at unclear risk of bias for incomplete outcome data since no reason for censoring was reported. They were at low risk in the reporting bias given both had research proposals and reported all the predetermined outcome indicators (primary and secondary outcomes). Regarding other bias, since no sufficient information to determine whether there was a risk of significant bias, we attributed them to unclear risk.

eFigure 6. Impacts of Key Factors on Incremental Cost-effectiveness Ratio

The diagrams show the impacts of key factors on the incremental cost-effectiveness ratio (Nivolumab versus Pembrolizumab) for the treatment of platinum-refractory R/M HNSCC. **A** represents the impacts of body weight, **B** represents the impacts of the cost of pembrolizumab, **C** represents the impacts of the cost of nivolumab, and **D** represents the impacts of HR for PFS (Nivolumab vs. Pembrolizumab). ICER= Incremental cost-effectiveness ratio; QALY= Quality-adjusted life year.

A

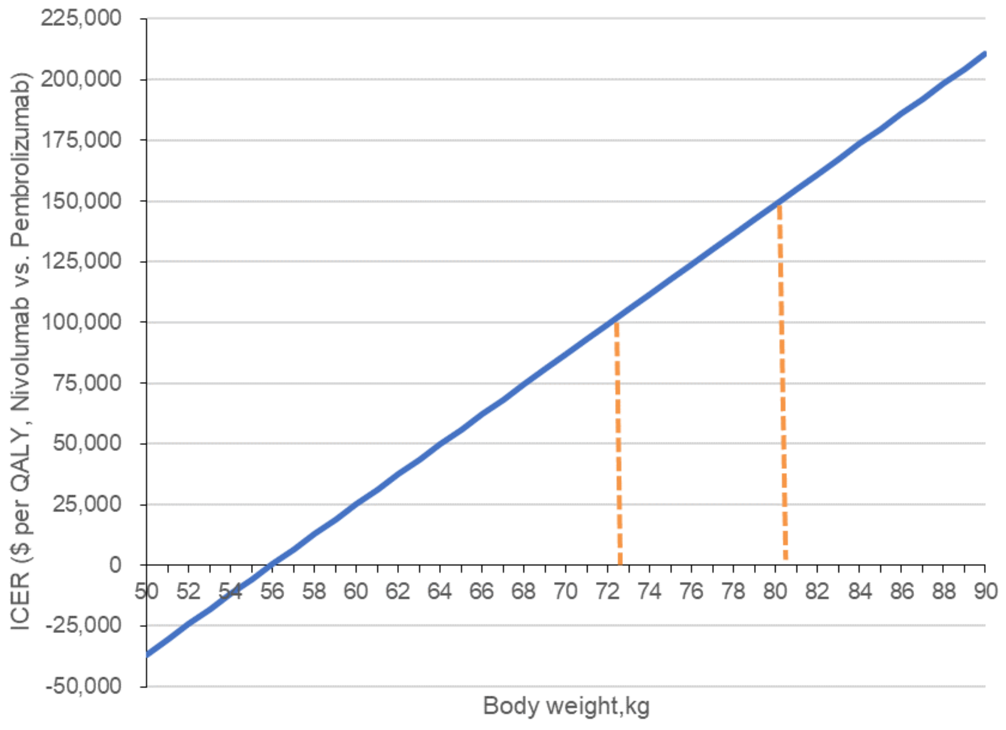

© 2021 Pei R et al. *JAMA Network Open*.

B

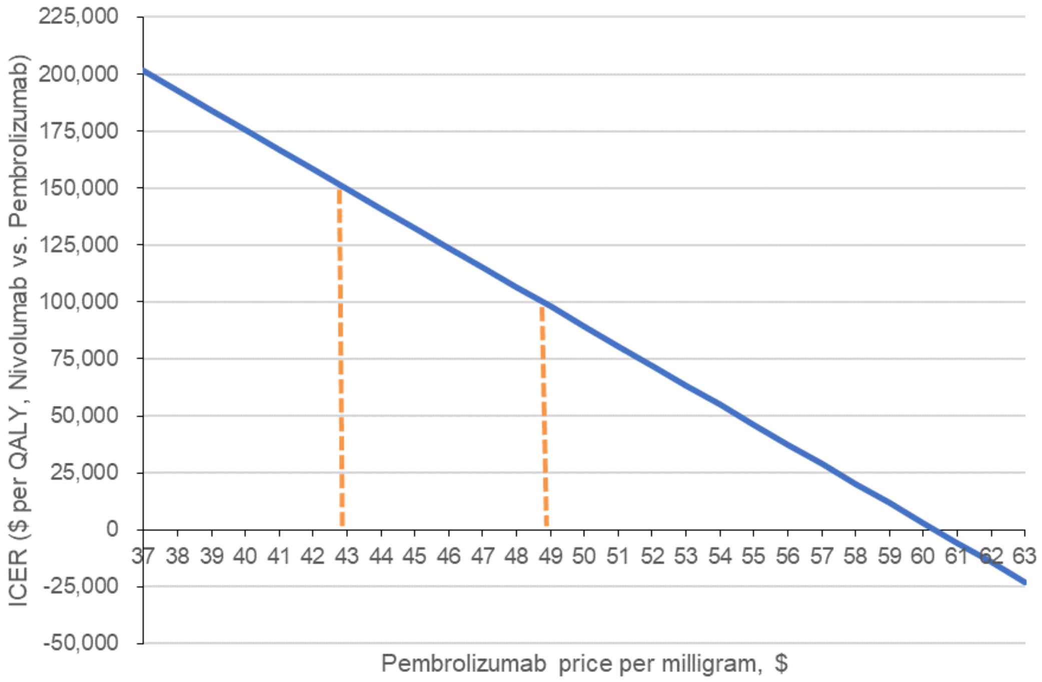
ICER ($ per QALY, Nivolumab vs. Pembrolizumab)
225,000
200,000
175,000
150,000
125,000
100,000
75,000
50,000
25,000
0
-25,000
-50,000
37 38 39 40 41 42 43 44 45 46 47 48 49 50 51 52 53 54 55 56 57 58 59 60 61 62 63
Pembrolizumab price per milligram, $

C

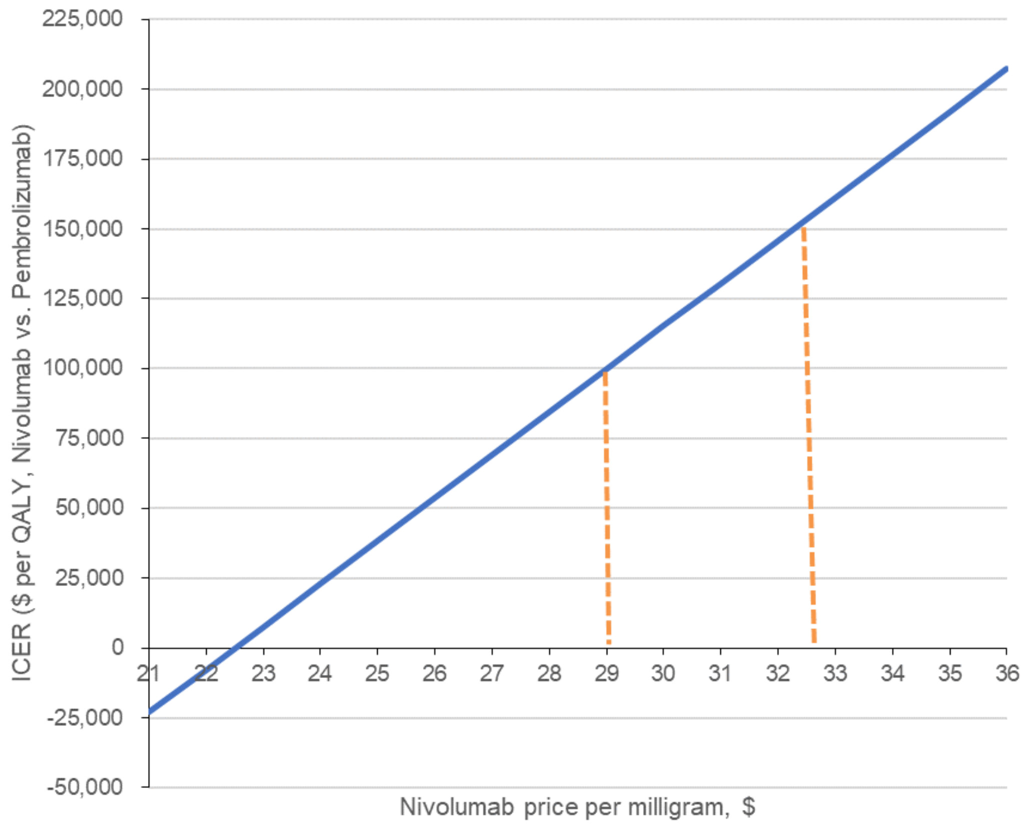

© 2021 Pei R et al. *JAMA Network Open*.

D

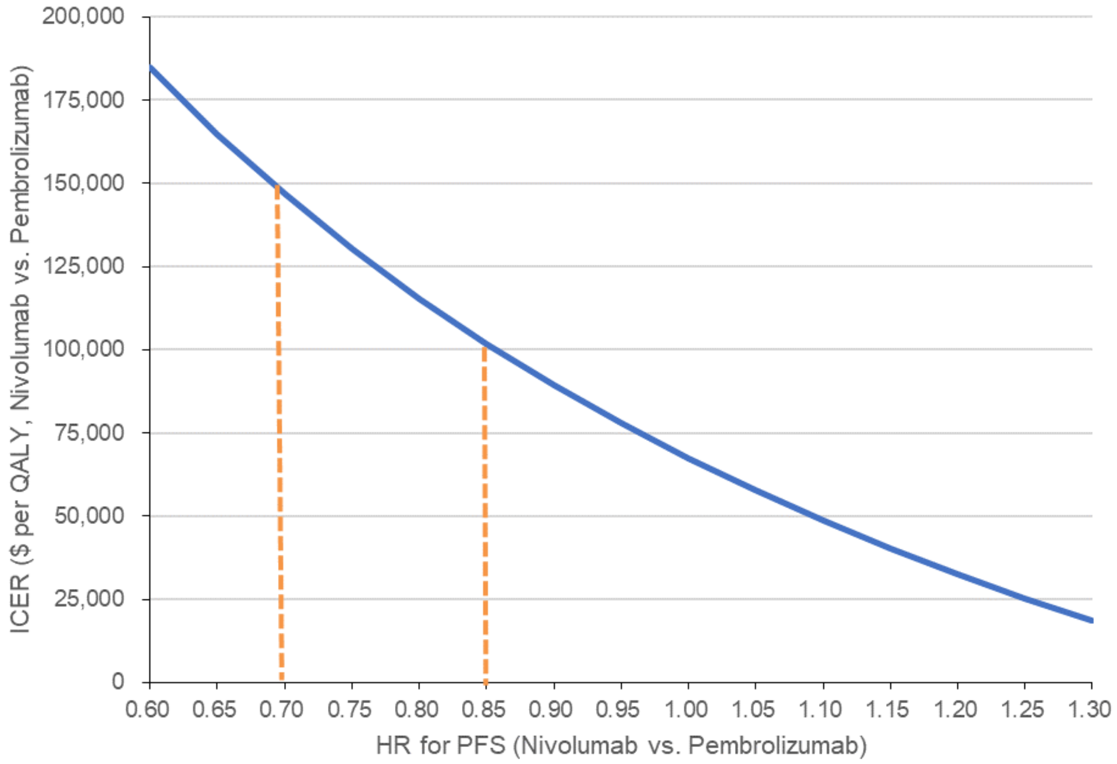

© 2021 Pei R et al. *JAMA Network Open*.

eFigure 7. Results of Two-Way Sensitivity Analyses

The following figures show the results of two-way sensitivity analyses for the cost of nivolumab and pembrolizumab (Net Benefit, **Figure A** represents WTP =$100,000/QALY, and **Figure B** represents WTP =$150,000/QALY). The threshold is the border between two regions. ICER= Incremental cost-effectiveness ratio, WTP= Willingness-to-pay, QALY= Quality-adjusted life year.

A

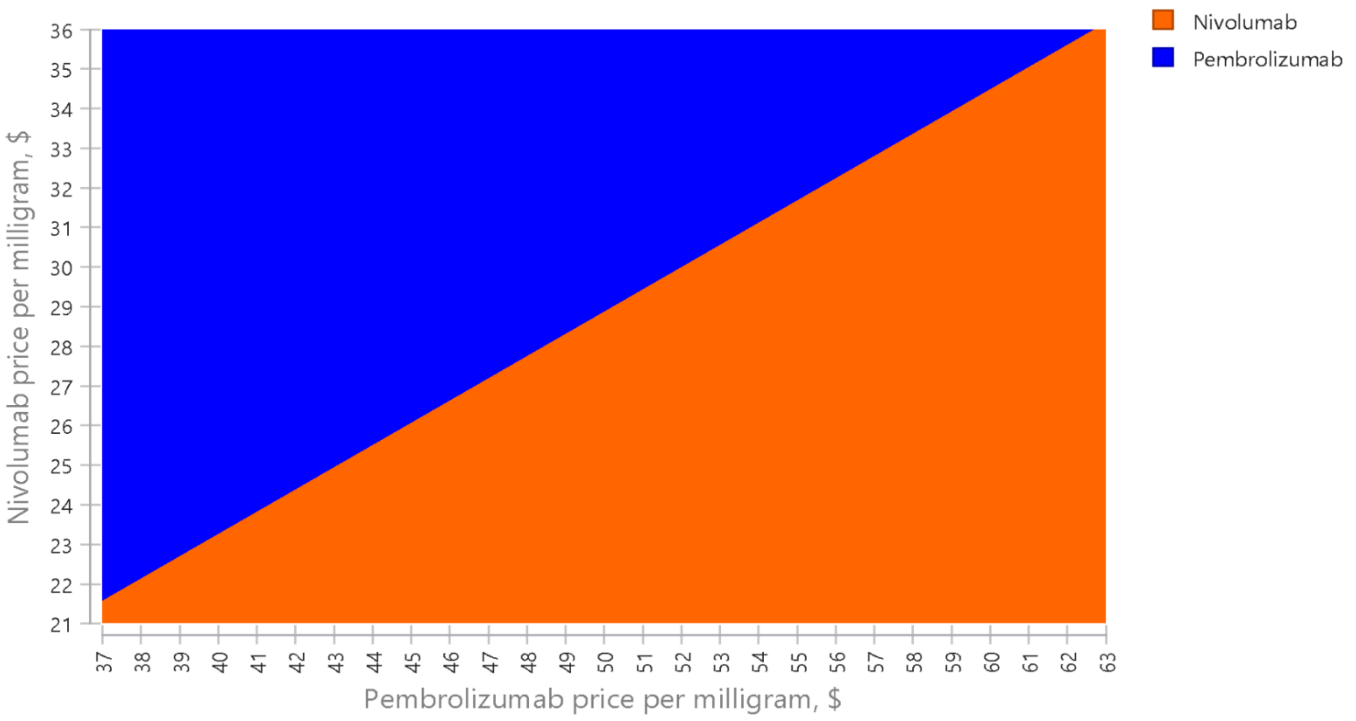

© 2021 Pei R et al. *JAMA Network Open*.

B

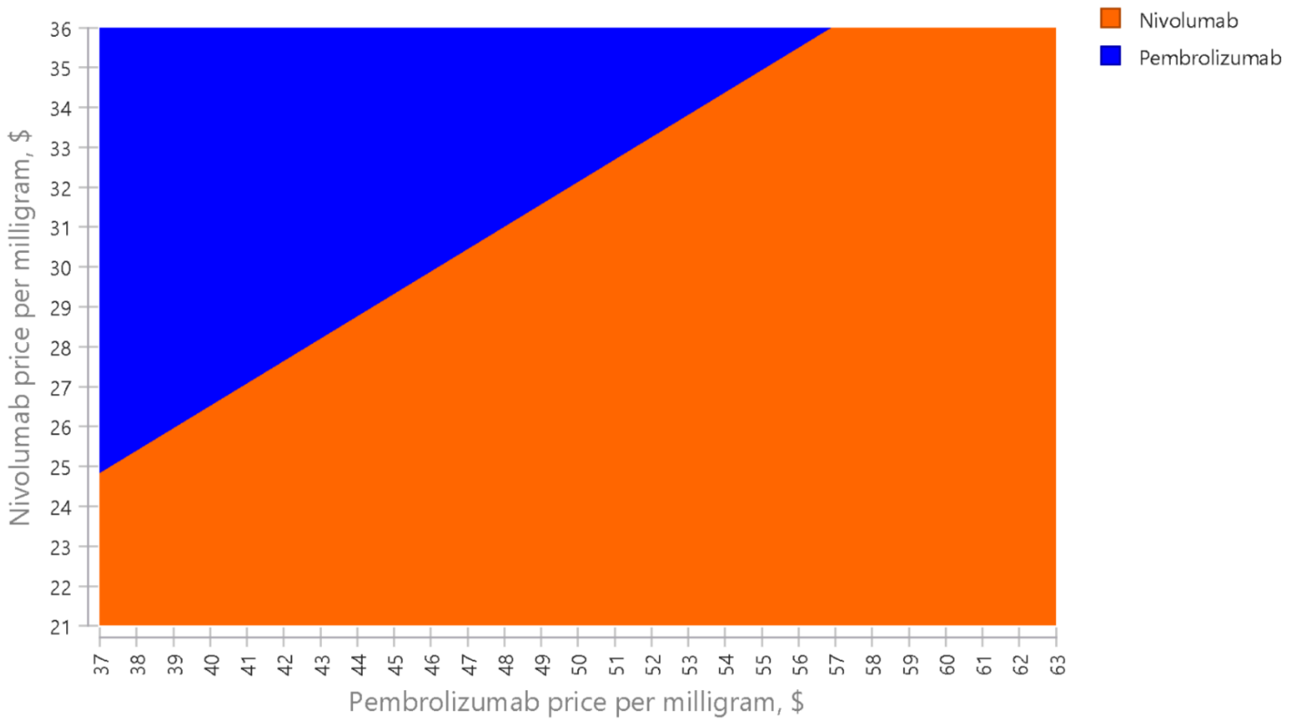
Nivolumab
Pembrolizumab
Nivolumab price per milligram, $
36
35
34
33
32
31
30
29
28
27
26
25
24
23
22
21
37
38
39
40
41
42
43
44
45
46
47
48
49
50
51
52
53
54
55
56
57
58
59
60
61
62
63
Pembrolizumab price per milligram, $

eFigure 8. Incremental Cost-effectiveness Scatter Plot (Nivolumab vs Pembrolizumab)

Plot A represents WTP =$100,000/QALY, and **Plot B** represents WTP =$150,000/QALY. ICER= Incremental cost-effectiveness ratio, WTP= Willingness-to-pay, QALY= Quality-adjusted life year.

A

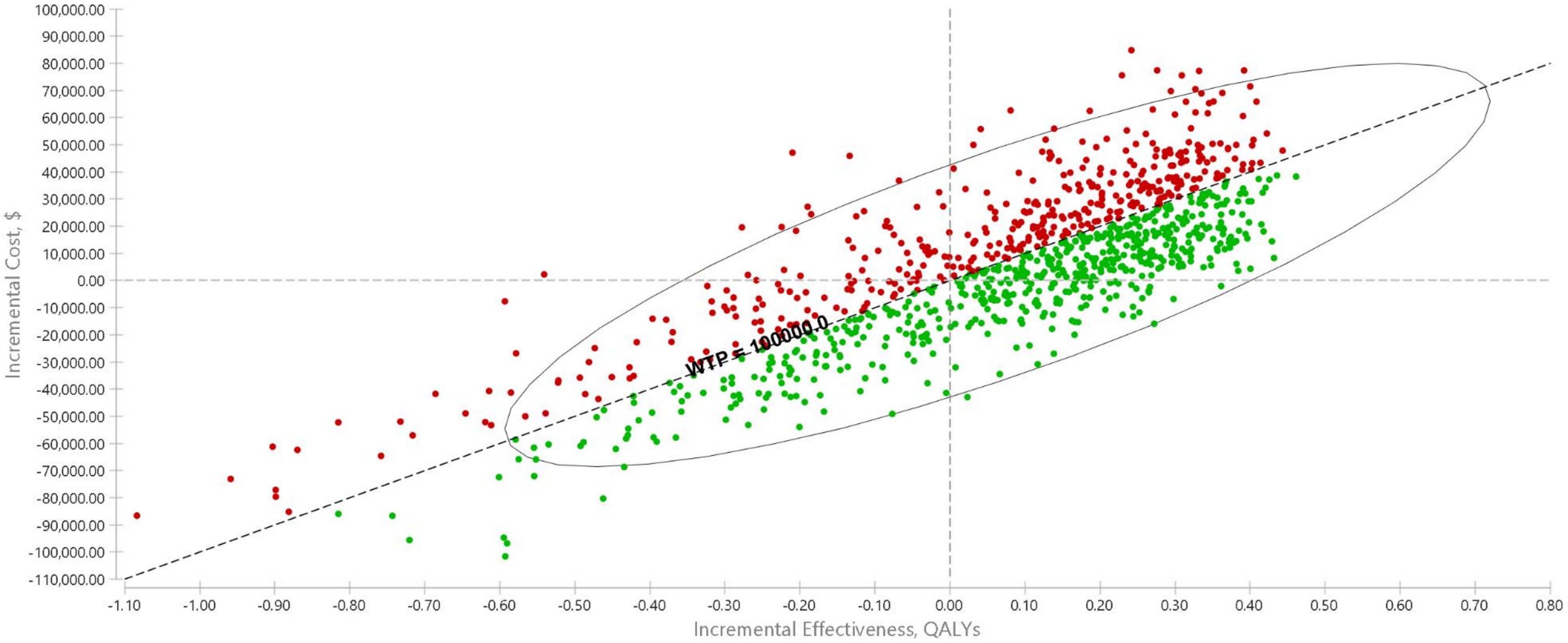

© 2021 Pei R et al. *JAMA Network Open*.

B

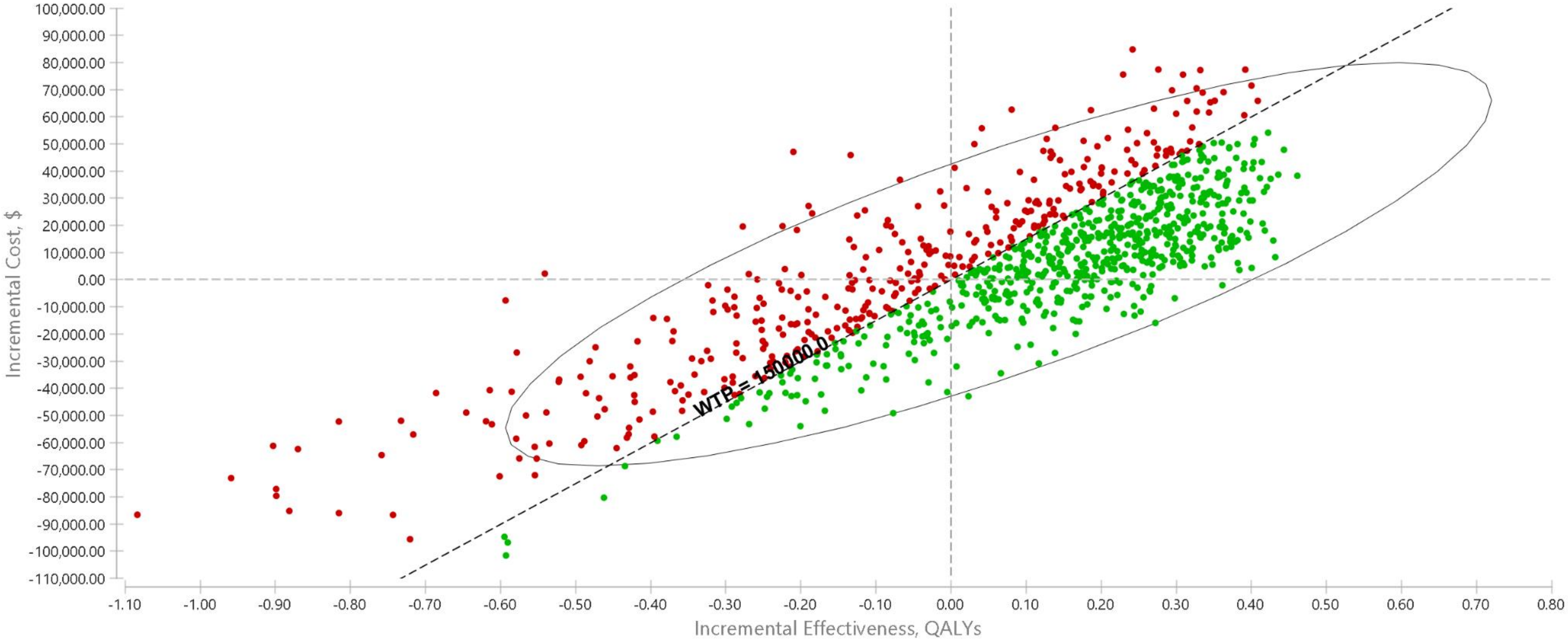

© 2021 Pei R et al. *JAMA Network Open*.

eMethods.

Study selection

We searched the Cochrane Central Register of Controlled Trials (CENTRAL), PubMed, and EMBASE for eligible publications until September 28, 2020 by using the strategy of (“MK-3475” OR "lambrolizumab" OR “pembrolizumab” OR “Keytruda” OR “BMS-936558” OR "MDX-1106" OR "ONO-4538" OR “nivolumab” OR “Opdivo”) AND ("HNC" OR "HNSCC" OR “SCCHN" OR (“head” AND “neck” AND ("carcinoma" OR "tumor" OR "cancer" OR "neoplasma" OR "malignancy"))). The eligible literature should meet the following criteria: (1) randomized controlled trials (RCTs); (2) compared pembrolizumab or nivolumab alone with chemotherapy for adult patients with platinum-refractory R/M HNSCC; and (3) the primary outcomes were OS or progression-free survival (PFS). Meeting abstracts in the American Society of Clinical Oncology (ASCO) and the European Society of Medical Oncology (ESMO) were also reviewed. When there were multiple reports about the same clinical trial, only the latest one was chosen. Publications written in non-English languages were excluded.

Subgroup analyses

Regarding subgroup analyses, at first, we conducted the network meta-analysis for subgroups based on the HRs (nivolumab versus mono-chemotherapy and pembrolizumab versus mono-chemotherapy) reported in two trials to obtain the HRs for OS (nivolumab versus pembrolizumab). Since the HRs for PFS in subgroups were unavailable, we assumed that the HR for PFS (nivolumab versus pembrolizumab) in subgroups was aligned with the total population, which was 0.91 (95% CI 0.66-1.25). Due to the lack of data in subgroups, other aspects such as the occurrence of SAEs were assumed to be consistent with the total population. The HRs for OS or PFS of each subgroup were then substituted into the model separately to make the cost-effective analysis.

© 2021 Pei R et al. *JAMA Network Open*.

eReferences.

[1] Haddad R, Cohen EEW, Venkatachalam M, et al. Cost-effectiveness analysis of nivolumab for the treatment of squamous cell carcinoma of the head and neck in the United States. *J Med Econ*. 2020;23(5):442-447.

[2] Cohen EEW, Soulières D, Le Tourneau C, et al. Pembrolizumab versus methotrexate, docetaxel, or cetuximab for recurrent or metastatic head-and-neck squamous cell carcinoma (KEYNOTE-040): a randomised, open-label, phase 3 study. *The Lancet*. 2019;393(10167):156-167.

[3] Centers for Medicare & Medicaid Services. 2020 ASP Drug Pricing Files. Accessed February 18, 2020. https://www.cms.gov/medicare/medicare-part-b-drug-average-sales-price/2020-asp-drug-pricing-files.

[4] Wolters Kluwer. UpToDate. Accessed March 25, 2020. https://www.uptodate.com.

[5] Centers for Medicare & Medicaid Services. 2020 Physician Fee Schedule Search. Accessed February 18, 2020. https://www.cms.gov/apps/physician-fee-schedule/search.

[6] Criss SD, Mooradian MJ, Sheehan DF, et al. Cost-effectiveness and Budgetary Consequence Analysis of Durvalumab Consolidation Therapy vs No Consolidation Therapy After Chemoradiotherapy in Stage III Non–Small Cell Lung Cancer in the Context of the US Health Care System. *JAMA Oncol*. 2019;5:358.

[7] Centers for Medicare & Medicaid Services. Addendum A and Addendum B Updates. Accessed March 3, 2020. https://www.cms.gov/Medicare/Medicare-Fee-for-Service-Payment/HospitalOutpatientPPS/Addendum-A-and-Addendum-B-Updates.

[8] Enomoto LM, Schaefer EW, Goldenberg D, Mackley H, Koch WM, Hollenbeak CS. The Cost of Hospice Services in Terminally Ill Patients With Head and Neck Cancer. *JAMA Otolaryngol--Head Neck Surg.* 2015;141:1066–1074.

[9] Gourin CG, Dy SM, Herbert RJ, et al. Treatment, survival, and costs of laryngeal cancer care in the elderly: Larynx Cancer Care in the Elderly. *The Laryngoscope*. 2014;124:1827–1835.

[10] Ward MC, Shah C, Adelstein DJ, et al. Cost-effectiveness of nivolumab for recurrent or metastatic head and neck cancer☆. *Oral Oncol.* 2017;74:49–55.

[11] Wong W, Yim YM, Kim A, et al. Assessment of costs associated with adverse events in patients with cancer. *PLoS ONE* 2018;13:e0196007.

[12] Hagiwara M, Borker R, Oster G. Economic Burden of Adverse Events in Patients With Metastatic Renal Cell Carcinoma. *Clin Ther.* 2013;35:1955-1963.e2.

[13] Nafees B, Lloyd AJ, Dewilde S, Rajan N, Lorenzo M. Health state utilities in non-small cell lung cancer: An international study. *Asia Pac J Clin Oncol.* 2017;13:e195–203.

[14] Kohn CG, Zeichner SB, Chen Q, Montero AJ, Goldstein DA, Flowers CR. Cost-Effectiveness of Immune Checkpoint Inhibition in BRAF Wild-Type Advanced Melanoma. *J Clin Oncol.* 2017;35:1194–202.

© 2021 Pei R et al. *JAMA Network Open*.

[15] Lloyd A, van Hanswijck de Jonge P, Doyle S, Cornes P. Health State Utility Scores for Cancer-Related Anemia through Societal and Patient Valuations. *Value Health*. 2008;11:1178–1185.

[16] Zargar M, McFarlane T, Chan KKW, Wong WWL. Cost−Effectiveness of Nivolumab in Recurrent Metastatic Head and Neck Squamous Cell Carcinoma. *The Oncologist.* 2018;23:225–233.

[17] Tam VC, Ko YJ, Mittmann N, et al. Cost-effectiveness of systemic therapies for metastatic pancreatic cancer. *Curr Oncol*. 2013;20:e90–106.

[18] Ferris RL, Blumenschein G, Fayette J, et al. Nivolumab for Recurrent Squamous-Cell Carcinoma of the Head and Neck. *N Engl J Med*. 2016;375:1856–1867.

[19] Ferris RL, Blumenschein G, Fayette J, et al. Nivolumab vs investigator's choice in recurrent or metastatic squamous cell carcinoma of the head and neck: 2-year long-term survival update of CheckMate 141 with analyses by tumor PD-L1 expression. Oral Oncol. 2018;81:45-51.

[20] Fukudo M, Ishikawa R, Mishima K, Ono T, Matsumoto S, Tasaki Y. Real-World Nivolumab Wastage and Leftover Drug Stability Assessment to Facilitate Drug Vial Optimization for Cost Savings. JCO Oncol Pract. 2020;16:e1134–1142.

[21] Sanders GD, Neumann PJ, Basu A, et al. Recommendations for Conduct, Methodological Practices, and Reporting of Cost-effectiveness Analyses: Second Panel on Cost-Effectiveness in Health and Medicine. JAMA. 2016;316:1093.

[22] Tringale KR, Carroll KT, Zakeri K, Sacco AG, Barnachea L, Murphy JD. Cost-effectiveness Analysis of Nivolumab for Treatment of Platinum-Resistant Recurrent or Metastatic Squamous Cell Carcinoma of the Head and Neck. *JNCI J Natl Cancer Inst.* 2018;110:479–485.

© 2021 Pei R et al. *JAMA Network Open*.
